# In Vitro Synergistic Interactions of Isavuconazole and Echinocandins against *Candida auris*

**DOI:** 10.3390/antibiotics10040355

**Published:** 2021-03-28

**Authors:** Unai Caballero, Sarah Kim, Elena Eraso, Guillermo Quindós, Valvanera Vozmediano, Stephan Schmidt, Nerea Jauregizar

**Affiliations:** 1 Department of Pharmacology, Faculty of Medicine and Nursing, University of the Basque Country (UPV/EHU), 48940 Leioa, Spain; unai.caballero@ehu.eus; 2 Center for Pharmacometrics and Systems Pharmacology, Department of Pharmaceutics, College of Pharmacy, University of Florida, Orlando, FL 32827, USA; sarahkim@cop.ufl.edu (S.K.); valva@cop.ufl.edu (V.V.); SSchmidt@cop.ufl.edu (S.S.); 3 Department of Immunology, Microbiology and Parasitology, Faculty of Medicine and Nursing, University of the Basque Country (UPV/EHU), 48940 Leioa, Spain; elena.eraso@ehu.eus (E.E.); guillermo.quindos@ehu.eus (G.Q.)

**Keywords:** *Candida auris*, checkerboard, combination, antifungal agents, isavuconazole, echinocandins, time-kill

## Abstract

*Candida auris* is an emergent fungal pathogen that causes severe infectious outbreaks globally. The public health concern when dealing with this pathogen is mainly due to reduced susceptibility to current antifungal drugs. A valuable alternative to overcome this problem is to investigate the efficacy of combination therapy. The aim of this study was to determine the in vitro interactions of isavuconazole with echinocandins against *C. auris*. Interactions were determined using a checkerboard method, and absorbance data were analyzed with different approaches: the fractional inhibitory concentration index (FICI), Greco universal response surface approach, and Bliss interaction model. All models were in accordance and showed that combinations of isavuconazole with echinocandins resulted in an overall synergistic interaction. A wide range of concentrations within the therapeutic range were selected to perform time-kill curves. These confirmed that isavuconazole–echinocandin combinations were more effective than monotherapy regimens. Synergism and fungistatic activity were achieved with combinations that included isavuconazole in low concentrations (≥0.125 mg/L) and ≥1 mg/L of echinocandin. Time-kill curves revealed that once synergy was achieved, combinations of higher drug concentrations did not improve the antifungal activity. This work launches promising results regarding the combination of isavuconazole with echinocandins for the treatment of *C. auris* infections.

## 1. Introduction

*Candida auris* (*C. auris*) is a multidrug-resistant yeast pathogen responsible for numerous cases of fungemia globally since 2009 [1]. This non-*Candida albicans* fungus spreads rapidly in hospitals and nursing homes, and it has been classified as an “urgent threat” pathogen according to the United States Centers for Disease Control and Prevention’s (CDC) 2019 Antibiotic Resistance Threats Report [2].

According to the first meeting of the WHO Antifungal Expert Group on Identifying Priority Fungal Pathogens, there is an overall consensus that *C. auris* is a pathogen of global public health interest and should be evaluated based on the limited existing therapeutic options due to resistance or other treatment issues [3].

Even though echinocandins are the recommended first-line treatment to fight *C. auris* infections [4], resistance to these drugs along with therapeutic failures have been reported [5]. Because of the limited available therapeutic options and the risk of treatment failure, alternative strategies, such as combination therapies, are being investigated. Recent works have evaluated the in vitro interactions of antifungal drugs against *C. auris* [6,7,8], or the combination of antifungal drugs with other antimicrobial agents [9,10,11,12]. Considering the scarce number of studies on antifungal combinations against *C. auris*, we aimed to further characterize the in vitro activity of the azole–echinocandin pairing against blood isolates of *C. auris*. Combining isavuconazole and echinocadins may be an interesting approach to be further investigated, considering that, on one hand, echinocandins are nowadays the treatment of choice for infections caused by *C. auris*, and on the other hand, isavuconazole is the newest and safer addition to the triazole group. Although it is labeled for the treatment of aspergillosis and mucormycosis, its anti-*Candida* activity together with its biopharmaceutical properties make it an interesting alternative [13]. In addition, the synergistic interactions obtained from the combination of isavuconazole plus anidulafungin against *C. auris* by the fractional inhibitory concentration index (FICI) method [14], and from isavuconazole plus micafungin against other non-*auris Candida* by time-kill studies [15], supports the interest of further studying the isavuconazole–echinocandin combinations against *C. auris*.

Therefore, the aim of this study was to examine the in vitro interactions and potential synergy between isavuconazole and the three currently commercialized echinocandins (anidulafungin, caspofungin, and micafungin) against *C. auris* clinical isolates. Drug interactions were first assessed by analyzing checkerboard data with non-parametric (FICI) and parametric (Greco and Bliss) approaches. Then, interaction results were verified by time-kill curve assays.

## 2. Results

### 2.1. Checkerboard Assays and Analysis

Checkerboard experiments showed that none of the antifungal drugs in monotherapy at the concentrations tested were able to stop fungal growth completely, expressed as an absorbance value close to 0%. Isavuconazole had a higher potency than echinocandins, as it reached a 50% reduction in the absorbance value at concentrations 0.06–0.125 mg/L, whereas echinocandins needed the highest concentrations tested (1–2 mg/L) to reach that threshold. Conversely, absorbance values close to 0% were reached with combinations that included ≥0.125 mg/L of isavuconazole and ≥0.5 mg/L of echinocandins. 

**Figure 1:**
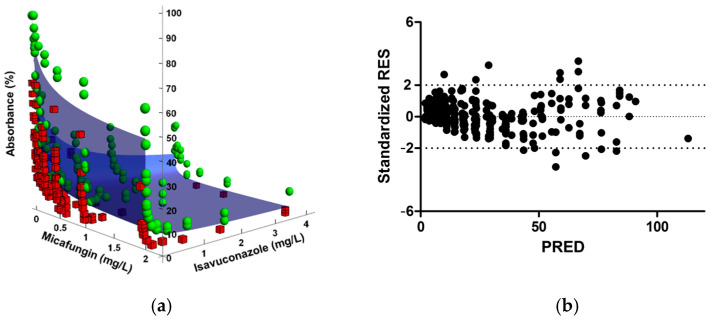
Goodness-of-fit plots of the Greco model for the combination of isavuconazole and micafungin against *C. auris* CJ100. (**a**) The blue surface represents model predictions, the green spheres represent observations above the fitted surface, and the red squares represent observations below the fitted surface. (**b**) Standardized residuals (Standardized RES) versus predictions (PRED).

**Figure 2:**
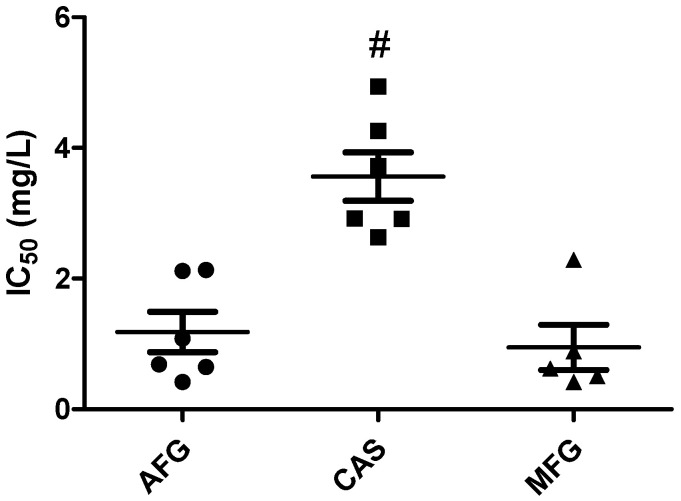
IC_50_ values determined by Greco model for each echinocandin and clinical strain. Mean and standard errors are plotted (#, *p* < 0.001 compared to anidulafungin (AFG) and micafungin (MFG)).

FICI results and the interaction parameters obtained from Greco and Bliss analysis for all *C. auris* isolates and drug combinations are summarized in Table 1. The determination of the FICI showed synergism for the three isavuconazole–echinocandin combinations against all isolates. On the other hand, synergistic interactions were found by the Greco model in 5 out of 6 clinical isolates for the combination of isavuconazole and micafungin, in 3 out of 6 for the combination of isavuconazole and anidulafungin, and in 2 out of 6 for the combination of isavuconazole and caspofungin. For the remaining isolates, the tested combinations were classified as additive, with a clear trend towards synergism, as shown by the 95% confidence interval (95% CI). Goodness-of-fit plots shown in Figure 1 for a representative isolate and drug combination revealed the concentrations for which the model-predicted effect (% absorbance) deviated from the experimental data. In general, the fitted response surface followed the same pattern as the experimental data, and no systematic deviation of the model was observed in the residual plots. However, the model deviated from the data at higher absorbance values, which corresponded to either echinocandin monotherapy or combinations with low concentrations of both drugs. As depicted in Figure 2, the IC_50_ obtained when fitting the Greco model to the experimental data was significantly higher for caspofungin than for anidulafungin or micafungin (*p* < 0.001).

**Figure 3:**
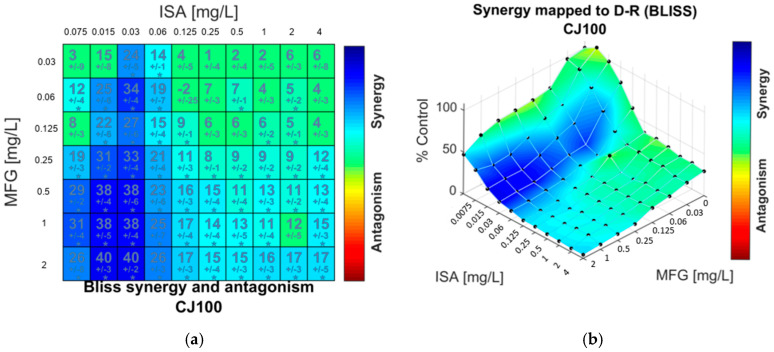
Synergy distribution determined by Bliss interaction model for the combination of isavuconazole (ISA) and micafungin (MFG) against *C. auris* CJ100. (**a**) Matrix synergy plot with synergy scores for each combination. (**b**) Synergy distribution mapped to dose–response surface.

The summary parameter values of the Bliss independence–based model, ΣSYN_ANT, showed weak synergistic interactions (values below 100%) for all combinations and isolates, except for isavuconazole and micafungin against *C. auris* CJ99 (Table 1). Both response surface methods, Greco and Bliss, were in concordance. An exception was isolate CJ97, as the combination of isavuconazole with anidulafungin or caspofungin was classified as synergistic with Greco model, but ΣSYN_ANT values were low (29.44 and 11.44%, respectively), and the distribution matrix showed a scarce number of synergic combinations. The median ΣSYN_ANT for combinations with caspofungin was lower than the ones for anidulafungin and micafungin (Table 1), but the difference was not statistically significant. Checkerboard results and Bliss analysis also revealed that both synergy and a low absorbance value effect were achieved with the combination consisting of low isavuconazole concentration (0.125 mg/L) and higher echinocandins (≥0.5 mg/L). The surface response according to the Bliss method of a representative isolate and drug combination in an 8 × 12 checkerboard design is depicted in Figure 3. A synergistic distribution and the degree of synergism are represented by the colored area.

### 2.2. Time-Kill Procedures

**Figure 4:**
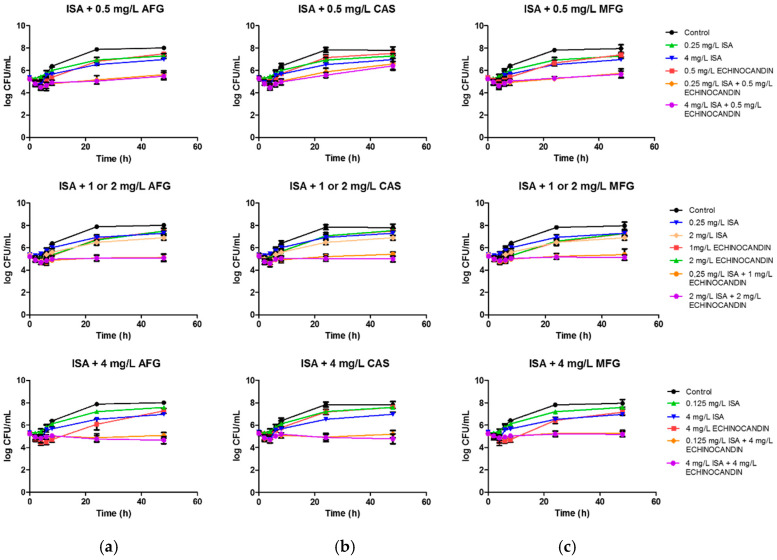
Mean time-kill curves for isavuconazole (ISA) in combination with echinocandins against *C. auris*. Each data point represents the mean result ± standard deviation (error bars) of the six isolates and replicates. (**a**) Mean time-kill curves for isavuconazole (ISA) in combination with anidulafungin (AFG). (**b**) Mean time-kill curves for isavuconazole (ISA) in combination with caspofungin (CAS). (**c**) Mean time-kill curves for isavuconazole (ISA) in combination with micafungin (MFG).

Mean time-kill curves for isavuconazole and echinocandins, alone and in combination, are shown in Figure 4. Drug monotherapies did not achieve significant antifungal activity, as observed in the figure and demonstrated by the positive k values (0.01–0.05 h^−1^). Conversely, synergism and fungistatic activity were achieved with combinations that included concentrations of isavuconazole ≥0.125 mg/L and echinocandin ≥1 mg/L, showing similar profiles of antifungal activity over time for all three azole–echinocandin combinations.

Although the activity of isavuconazole plus 0.5 mg/L of anidulafungin or micafungin was not fungistatic according to the established definition, the regression analysis of the curves revealed that the killing-rate constant of those drug combinations was not significantly different from a zero slope, indicating a lack of fungal growth through 48 h. That was not the case for the combinations with 0.5 mg/L of caspofungin, as it did not result in a significant reduction in fungal growth (positive k value of 0.02 h^−1^). This result correlates with the aforementioned Greco analysis that pointed out a lower potency for this echinocandin. Combinations that included concentrations of echinocandin ≥1 mg/L also yielded curves with a killing-rate constant non-different from zero, indicating that once the fungistatic effect was achieved, increasing drug concentrations for both agents did not result in a significant reduction in fungal count over time (see also Table S1, Supplementary Materials).

## 3. Discussion

Among the different strategies to fight and overcome antimicrobial resistance, combination therapy is an attractive approach. This alternative strategy is increasingly interesting for the treatment of candidiasis caused by *C. auris,* recently classified as an “urgent threat” pathogen and with limited treatment choices [2].

In the present study, isavuconazole and echinocandin combinations showed promising results, as they were deemed mainly synergistic by different analytical approaches and were able to halt fungal growth for 48 h in time-kill experiments. Furthermore, the synergism resulting from this combination therapy is particularly relevant if we consider the lack of efficacy shown by the studied drugs in monotherapy. We found similarities between our monotherapy results and those reported by Dudiuk et al. [16] for *C. auris*, as no fungicidal activity was achieved with the echinocandins against the tested *C. auris* strains while similar time-kill curve patterns were observed. The in vitro evidence of non-fungicidal activity of the drugs in monotherapy supports the interest to study drug combinations. Up to now, there are only seven published studies that have evaluated the in vitro activity of antifungal drug combinations against *C. auris*. In a recent study, Pfaller et al. [14] used the FICI analysis to examine the in vitro activity of voriconazole or isavuconazole in combination with anidulafungin against *C. auris* isolates. They observed synergism or partial synergism against most isolates, greater for the combination of isavuconazole plus anidulafungin compared to voriconazole plus anidulafungin. In a previous study, Fakhim et al. [6] reported that voriconazole and micafungin exhibited synergism against *C. auris* determined by the FICI, whereas the combinations of voriconazole with caspofungin or echinocandins with fluconazole resulted indifferent. Therefore, the synergy results of our study are consistent with these two aforementioned reports. Furthermore, we validated the checkerboard results with time-kill experiments.

O’Brian et al. [8] studied *C. auris* isolates from a New York outbreak and found synergism for the combination of flucytosine with the rest of antifungal classes, but not for the combination of azoles with echinocandins. Conversely, flucytosine showed no interaction with other antifungal drugs against Indian *C. auris* isolates [7]. This highlights the fact that the antimicrobial activity of drug–drug interactions may be not only species specific but also strain specific. Other works have focused on the combination of antifungal drugs with non-antifungal agents as an approach to enhance the therapeutic arsenal against *C. auris* [9,10,11,12]. To date, the only published in vivo combination study, a model of *Caenorhabditis elegans* infected with *C. auris*, supported the combination of sulfamethoxazole and voriconazole [9]. However, there is no in vivo or clinical evidence regarding the combination of echinocandins plus azoles against *C. auris*. Additionally, *C. auris* is able to form biofilms on the skin, on implanted medical devices or in the hospital environment [17]. As these biofilms are even more resistant to antifungal agents compared to planktonic cells [17,18], the possible benefits of the aforementioned synergy should also be assessed in fighting such a biofilm community.

Synergism applied to drug–drug combinations can be defined in a simple way as the interaction between two or more compounds that exerts a greater effect than the additive sum of the effects of each drug when acting alone [19]. Nevertheless, determination of synergism or antagonism is far from simple. There are many factors that have to be taken into account, such as the experimental setting to obtain the empirical data or the mathematical methods chosen for the analysis [20]. Widely accepted and employed theories for the determination of drug interactions are Loewe’s additivity and Bliss independence [21]. When analyzing checkerboard data of antimicrobial drugs, Loewe’s additivity is usually determined by the non-parametric approach of the FICI, obtained by comparing the MIC of the compounds alone and in combination [22]. On one hand, the FICI method is well established and straightforward; on the other, it ignores all the concentration–response data that do not correspond to MICs, and variable results and interpretations may be expected depending on the MIC endpoints [23]. Parametric approaches, such as the Greco universal response surface approach (URSA) and the Bliss interaction model, overcome this drawback, as the whole drug-concentration range is analyzed [24]. It also allows the estimation of parameters (such as IC_50_) and the associated confidence intervals based on more robust mathematical and statistical methods [25]. The results obtained with the parametric and non-parametric approaches showed synergistic interactions for the isavuconazole–echinocandin combinations. Furthermore, this agreement between approaches and the sensitivity of the models in detecting even weak interactions is concordant with other reports that have compared different drug interaction models [10,23,26].

It is important to take into consideration that a lack of synergism of a drug combination does not necessarily mean that the effectiveness of the combination is negligible. After all, when antimicrobial agents are combined, the goal is to reduce the microbial density [27], regardless of the nature of the interaction. Additionally, for the interpretation of a synergism result, it should be considered whether it has been observed under clinically relevant conditions, whether the observed synergism has been obtained at concentrations that are clinically achievable, and the extent of the effect. Bliss independence model based surface analysis allowed us to identify the concentration range of each combined drug where synergism was claimed. In summary, synergism was detected for the combination of low concentrations (<0.125 mg/L) of both isavuconazole and echinocandins. The antifungal effect was further examined by time-kill experiments in which we selected the concentrations that showed zero absorbance value in the checkerboard assays, as that reflects an antifungal effect of interest. It was observed that fungal growth was reduced over 48 h by all the combinations tested, but none of them achieved fungicidal activity. Furthermore, the time-kill curve analysis revealed that once the fungistatic effect and synergism were achieved with the echinocandin–azole combinations, higher concentrations did not result in a higher reduction in fungal burden. These threshold concentrations were isavuconazole ≥0.125 mg/L and echinocandin ≥1 mg/L. Time-kill curve experiments also supported that an additive effect can be of interest, as none of the drugs in monotherapy showed fungistatic activity, while the interaction of all combinations at 24 h was additive but also fungistatic. To our knowledge, this is the first study that has determined the in vitro interactions of a triazole with echinocandins against *C. auris* by use of both checkerboard and time-kill assays.

The main limitation of the present study is the limited number of isolates tested. This limitation has also been recognized in previous studies of antifungal drug interactions against *C. auris* [14]. Moreover, our study only included bloodstream isolates of *C. auris*, collected from the main *C. auris* outbreak in a Spanish hospital, first detected in 2016.

It is worth noting that the antifungal effect and the synergy observed with the studied combinations were observed at clinically achievable and safe drug concentrations related to standard dosing [28]. Nevertheless, any in vitro system, no matter how sophisticated, is a simplification of the much more complex in vivo situation [29], and proper in vitro–in vivo correlations have not been established for azole–echinocandin combinations. In fact, in vivo and clinical studies on antifungal combination therapy in *C. auris* infections are lacking. The results of this work could assist in the design of further in vivo and clinical studies to better asses the therapeutic use of isavuconazole–echinocandin combination.

## 4. Materials and Methods

### 4.1. Fungal Strains

Six *C. auris* clinical blood isolates (CJ94, CJ97, CJ98, CJ99, CJ100, and CJ102) from an outbreak in the Hospital Universitario y Politécnico La Fe (Valencia, Spain) [30] were included in this study. Fungal strains were stored in vials with sterile distilled water at room temperature for up to 1 year, while commercially prepared cryogenic Microbank vials (Pro-Lab Diagnostics, USA) maintained at −70 °C were used for prolonged storage. Strains were cultivated in Sabouraud dextrose agar (SDA) for 24 h before every experiment. MIC determination at 24 h was performed following EUCAST guidelines [31]. MICs for isavuconazole, anidulafungin, caspofungin, and micafungin were 0.06, 0.125, 0.125, and 0.25 mg/L, respectively, for all isolates.

### 4.2. Antifungal Agents

Isavuconazole (Basilea Pharmaceutica International, Basel, Switzerland), anidulafungin (Pfizer SLU, Madrid, Spain), caspofungin (Merck Sharp & Dohme, Madrid, Spain), and micafungin (Astellas Pharma, Madrid, Spain) were obtained in powder form and were prepared and preserved according to their respective manufacturer’s recommendations. The drugs were dissolved in dimethyl sulfoxide (DMSO) to obtain stock solutions of 3200 mg/L and stored at −80 °C until use.

### 4.3. Checkerboard Assay

Interactions between isavuconazole and echinocandins were first studied by a checkerboard microdilution method (8 × 12 design) in 96-well flat-bottom microtiter plates, in RPMI 1640 medium, following EUCAST guidelines and modified for drug combinations [7,10]. Isavuconazole was added to columns 2–11, with concentrations ranging from 0.0075 to 4 mg/L. Aliquots of anidulafungin, caspofungin, or micafungin were added to rows A–G, with a range of 0.03–2 mg/L. Thus, concentrations of the drug combinations ranged from 0.0075 mg/L of isavuconazole + 0.03 mg/L of echinocandin to 4 mg/L of isavuconazole + 2 mg/L of echinocandin. Wells from column 12 were left for growth control, and wells H1 and H12 were used as sterility control. On the day of the experiment, *C. auris* isolates, previously incubated at 37 °C overnight, were suspended in distilled water to obtain a starting inoculum of 0.5–2.5 × 10^5^ CFU/mL and were added to the microtiter plates. Those plates were then incubated at 37 °C for 48 h, and the absorbance of each well was measured on an Infinite F50 spectrophotometer (Tecan, Switzerland) at a wavelength of 450 nm. *Candida parapsilosis* ATCC 22019 and *Candida krusei* ATCC 6258 were used as quality controls. Experiments were conducted in triplicate on different days.

### 4.4. Data Analysis

Absorbance data were transformed into percentages, with the mean value of the growth control absorbance set to 100%. The data were analyzed by FICI [22] and surface response models: Loewe’s additivity–based Greco model and Bliss independence [23,24].

#### 4.4.1. FICI

The determination of the FICI of a drug combination is based on Loewe’s additivity and is calculated as follows:

(1) $$\mathrm{FICI}=\frac{{\mathrm{MIC}}_{A+B}}{{\mathrm{MIC}}_{A}}+\frac{{\mathrm{MIC}}_{A+B}}{{\mathrm{MIC}}_{B}}$$

where MIC_A+B_ is the MIC of the drugs A and B in combination, MIC_A_ the MIC of drug A alone, and MIC_B_ the MIC of drug B alone. Drug interactions were defined as synergistic if FICI ≤ 0.5, no interaction if FICI > 0.5 ≤ 4, and antagonistic if FICI > 4 [22].

#### 4.4.2. Greco Model

The parametric surface approach described in the Greco model is defined in the following equation:

(2) $$1=\frac{{\mathrm{Drug}}_{1}}{IC_{50,1}\times {\left(\frac{E}{E_{\mathrm{con}}-E}\right)}^{\left(\frac{1}{m1\;}\right)}}+\frac{{\mathrm{Drug}}_{2}}{IC_{50,2}\times {\left(\frac{E}{E_{\mathrm{con}}-E}\right)}^{\left(\frac{1}{m2}\right)}}+\frac{\alpha \times {\mathrm{Drug}}_{1}\times {\mathrm{Drug}}_{2}}{IC_{50,1}\times IC_{50,2}\times {\left(\frac{E}{E_{\mathrm{con}}-E}\right)}^{\left(\frac{1}{2m1\;}+\frac{1}{2m2\;}\right)}}\;$$

where Drug_1_ and Drug_2_ are the concentrations of isavuconazole and echinocandin, respectively, IC_50,1_ and IC_50,2_ are the concentrations of each drug that achieve 50% of the maximum activity, m1 and m2 are the slopes of the concentration–effect curves or Hill’s coefficient, E_con_ is the effect in the absence of drug, E is the fractional effect, and α is the interaction parameter that describes the nature of the interaction. When the value of α was positive and its 95% confidence interval (95% CI) was also positive, the interaction was defined as synergistic. A positive α with 95% CI including zero described an additive interaction. When α was negative without its 95% CI overlapping zero, an antagonistic interaction was claimed. The analysis was run in ADAPT 5 [32], with weighted least-squares method and three-dimensional goodness-of-fit plots generated by Mathematica (v 12.1; Wolfram Research Inc., Champaign, IL, USA). The parameters obtained for the different combinations were then compared by one-way ANOVA and Tukey’s multiple comparison test using GraphPad Prism 5.01 (GraphPad Software, San Diego, CA, USA).

#### 4.4.3. Bliss Independence Model

The Bliss independence model, which assumes that the relative effect of a drug at a particular concentration is independent of the other drug, is defined by the following equation:

ΔE = E_ind_ − E_obs_ (3)

where ΔE is the difference between the predicted percentage of growth (E_ind_) and the observed percentage of growth (E_obs_). E_ind_ is calculated, in turn, as follows:

E_ind_ = E_A_ × E_B_ (4)

where E_A_ and E_B_ are the observed percentage of growth in the presence of drug A and drug B, respectively.

When the ΔE of each specific combination of *x* mg/L of isavuconazole and *y* mg/L of echinocandin was positive and its 95% CI did not include zero, the interaction was defined as synergistic. When the ΔE was negative and its 95% CI did not include zero, the interaction was defined as antagonistic. Any other case was considered indifferent.

The sum of all statistically significant synergistic and antagonistic interactions (ΣSYN_ANT) was considered as the main parameter that summarized the whole interaction surface for the studied three combinations [15,26]. Additionally, when the ΣSYN_ANT value obtained for each checkerboard analysis was below 100%, weak interaction was defined, between 100% and 200% was considered moderate, and those higher than 200% were considered as strong [15,23]. Combenefit was the software used to perform the Bliss analysis [33].

### 4.5. Time-Kill Procedures

Static time-kill assays were performed as previously described [34] and adapted with minor modifications for antifungal combinations. The concentrations of the drugs selected for the study were based on the checkerboard results. Isavuconazole concentrations were 0.125, 0.25, 2, and 4 mg/L, and anidulafungin, caspofungin, or micafungin concentrations ranged from 0.5 to 4 mg/L. Inoculum preparation was carried out as in the checkerboard assay previously described, but with a final fungal density of 1–5 × 10^5^ CFU/mL. Samples for viable counts were taken at 0, 2, 4, 6, 8, 24, and 48 h, diluted in PBS, plated in triplicate onto Sabouraud dextrose agar (SDA), and incubated for 24–48 h at 37 °C to determine the number of CFU/mL. Fungistatic activity was defined as a reduction <3 log CFU/mL compared to the starting inoculum. Synergism was declared when the difference between the drug with highest effect in monotherapy and the combination was >2 log CFU/mL [35]. Experiments were performed in duplicate on different days. Time-kill curves were analyzed by fitting the observations to the following exponential equation, as previously described [34]:

N_t_ = N_0_ × e^kt^ (5)

where N_t_ is the number of CFU/mL at time t, N_0_ is the starting inoculum, k is the growing or killing-rate constant, and t is the incubation time. Positive k values show growth, and negative values indicate killing. Goodness-of-fit for each combination was assessed by the r^2^ value (>0.8). Significant differences in killing kinetics among combinations and concentrations were determined by the analysis of variance. A *p* value < 0.05 was considered significant.

## 5. Conclusions

In conclusion, in the present in vitro study, the combinations of isavuconazole with anidulafungin, caspofungin, or micafungin against *C. auris* were mostly synergistic and fungistatic, providing evidence that combination therapy is a promising approach to be further investigated, especially when the drugs alone show reduced activity against *C. auris*.

## Figures and Tables

**Table 1:** Fractional inhibitory concentration index (FICI) and interaction parameters determined by Greco model (α) and Bliss model (ΣSYN_ANT) ^a^.

*C. auris* isolate	ISA + AFG ^b^			ISA + CAS ^b^			ISA + MFG ^b^		
	FICI	Greco	Bliss	FICI	Greco	Bliss	FICI	Greco	Bliss
	Median (Range)	α (95% CI)	ΣSYN_ANT (ΣSYN; ΣANT)	Median (Range)	α (95% CI)	ΣSYN_ANT (ΣSYN; ΣANT)	Median (Range)	α (95% CI)	ΣSYN_ANT (ΣSYN; ΣANT)
CJ94	0.27 (0.24–0.30)	151 (16.53–285.5)	86.91 (87.22; −0.31)	0.26 (0.25–0.52)	38.59 (−8.106–85.28)	36.56 (41.22; −4.66)	0.24 (0.15–0.38)	112.8 (22.70–202.9)	66.01 (66.79; −0.78)
CJ97	0.36 (0.25–0.49)	21.70 (5.105–38.38)	29.24 (30.27; −1.03)	0.37 (0.36–1.25)	22.23 (1.016–43.44)	11.44 (20.75; −8.56)	0.15 (0.13–0.25)	216.7 (11.37–422.0)	57.59 (59.49; −1.90)
CJ98	0.19 (0.015–0.19)	102.1 (4.056–200.1)	57.38 (57.73; −0.35)	0.25 (0.08–0.5)	42.64 (−14.72–100.00)	40.67 (45.46; −4.79)	0.37 (0.15–0.49)	57.88 (0.85–114.9)	50.88 (53.70; −2.82)
CJ99	0.18 (0.09–0.18)	186.9 (−3.962–377.8)	73.23 (73.32; −0.09)	0.25 (0.18–0.37)	114.4 (−1.911–230.6)	75.71 (75.89; −0.18)	0.16 (0.08–0.38)	674.4 (−138.3–1487)	111.56 (112.09; −0.53)
CJ100	0.18 (0.15–0.37)	48.71 (−15.71–113.1)	72.80 (75.17; −2.37)	0.38 (0.25–0.49)	37.12 (2.344–71.90)	60.31 (60.64; −0.33)	0.14 (0.12–0.15)	175.1 (14.32–335.8)	80.14 (80.37; −0.23)
CJ102	0.25 (0.14–0.36)	204.1 (−5.106–413.9)	69.61 (69.63; −0.02)	0.36 (0.25–0.38)	41.85 (−1.016–84.71)	46.37 (48.08; −1.71)	0.25 (0.13–0.49)	95.66 (24.84–166.5)	71.35 (71.72; −0.37)
Median	0.22	–	71.205	0.31	–	43.52	0.20	–	68.68

^a^ Synergic interactions according to FICI and Greco model are underlined. ΣSYN_ANT: total sum of synergic and antagonistic interactions. ΣSYN: sum of synergic interactions. ΣANT: sum of antagonistic interactions. ^b^ ISA, isavuconazole; AFG, anidulafungin; CAS, caspofungin; MFG, micafungin.

## Data Availability

Not applicable.

## Supplementary Materials

The following are available online at https://www.mdpi.com/article/10.3390/antibiotics10040355/s1, Table S1: Average killing rate constant (k) values for the monotherapies and drug combinations against *Candida auris*.
