# Supplementary material for: In Vitro Synergistic Interactions of Isavuconazole and Echinocandins against Candida auris
Source: Antibiotics (Basel). 2021 Mar 28;10(4):355. doi: 10.3390/antibiotics10040355 (PMC8066733; doi:10.3390/antibiotics10040355)
Supplement: Supplementary file 1 [file antibiotics-10-00355-s001.pdf]

Table S1: Average killing rate constant (k) values for the monotherapies and drug combinations against *Candida auris*.

	k of Monotherapies (h^{-1})		
Control	0.07		
	**ISA**[b]		
0.125 mg/L	0.05		
0.25 mg/L	0.048		
2 mg/L	0.028		
4 mg/L	0.034		
	**AFG**[b]	**CSP**[b]	**MFG**[b]
0.5 mg/L	0.015	0.034	0.011
1 mg/L	0.01	0.032	0.01
2 mg/L	0.01	0.031	0.011
4 mg/L	0.01	0.039	0.01
	**k of Combinations (h^{-1})**		
**ISA + Echinocandin**	**ISA + AFG**[b]	**ISA+ CAS**[b]	**ISA + MFG**[b]
0.25 mg/L + 0.5 mg/L	0	0.025	0
4 mg/L + 0.5 mg/L	0	0.02	0
0.25 mg/L + 1 mg/L	0	0	0
2 mg/L +2 mg/L	0	0	0
0.125 mg/L + 4 mg/L	0	0	0
4 mg/L + 4 mg/L	0	0	0

[b]ISA, isavuconazole; AFG, anidulafungin; CAS, caspofungin; MFG, micafungin
